# In the ICU and post ICU, platelets have more impact as a prognosis marker than leukocytes!

**DOI:** 10.1186/cc14707

**Published:** 2015-09-28

**Authors:** Carlos Augusto R Feijó, Allison EPP Borges, Eduardo Q da Cunha, Francisco A de Meneses, Marina P Albuquerque, Natália LP Aragão, Tamara O Pinheiro, Túlio S de Aguiar

**Affiliations:** 1 General Hospital of Fortaleza, Papicu, Fortaleza, CE, Brazil

## Introduction

In everyday practice, intensivists constantly search for tools to infer the prognosis of patients. The most simple and feasible markers stand out before the most complex and expensive ones. Traditionally, the white blood cell (WBC) count has been used as an inflammatory discriminant, rather than the platelet count. A comparison between these two parameters as predictors of ICU patient outcome, however, has been recently emphasized.

## Objective

To correlate the levels of leukocytes, platelets, and the platelets/leukocytes ([P/L]) ratio with patient outcome, as far as discharge/death in the ICU or in-hospital, concerning patients admitted to the ICU.

## Methods

Data from patients admitted to the General Hospital of Fortaleza's ICU, from November 2014 to February 2015, were retrospectively analyzed. Complete blood count (CBC) data analyzed were collected on the first (D1) and fifth (D5) ICU days. Statistical analysis included the *t *test for evaluation of the WBC count, platelet count and [P/L] ratio relative to patient outcome, meaning ICU and in-hospital discharge/death. To compare these contents, the area under the ROC curve was computed for each index on D1 and D5. Patients with incomplete data were excluded from the study.

## Results

From a total of 86 patients, 51 % were male, mean age was 53 ± 19 years and mean APACHE II score was 14 ± 7 points. CBC assessment demonstrated that the mean platelet number was significantly lower between patients who died and those who were discharged from the ICU and hospital, both on D1 and D5 (platelet count performed well in discriminating the ICU (AUC = 0.831) and hospital (AUC = 0.81) outcome). Despite the poor performance on D1, the D5 [P/L] ratio had regular performance for hospital outcome (AUC = 0.752), and good for ICU outcome (AUC = 0.803).

## Conclusion

These findings suggest that continued assessment of CBC, especially platelets and the [P/L] ratio, has better performance than leukocytes to infer the outcomes of the ICU and, subsequently, the hospital.

